# Variations in colorectal cancer pattern of care by age and comorbidity in South Australia

**DOI:** 10.1002/cam4.5901

**Published:** 2023-04-21

**Authors:** Kazzem Gheybi, Elizabeth Buckley, Agnes Vitry, David Roder

**Affiliations:** ^1^ University of South Australia Allied Health and Human Performance South Australia Adelaide Australia; ^2^ Cancer epidemiology and population health University of South Australia South Australia Adelaide Australia; ^3^ Charles Perkins Centre, School of Medical Sciences University of Sydney New South Wales Sydney Australia; ^4^ University of South Australia Clinical and Health Sciences South Australia Adelaide Australia

**Keywords:** advanced age, chemotherapy, comorbidity, surgery, treatment variation

## Abstract

**Background:**

Advanced age is associated with decreased likelihood of colorectal cancer treatment. Here, we investigated the extent to which comorbidities are accountable for this lesser treatment.

**Methods:**

Using population‐based datasets, the pattern of care among CRC cases in South Australia during 2004–2013 was investigated. Models were used to investigate associations of age with each treatment type, and differences in these associations were explored by comorbidity and cancer site.

**Results:**

The presence of comorbidity was associated with a significantly weaker relationship of age with surgery and chemotherapy. The association of age with surgery also varied for colon and rectal primary cancer sites. Individual comorbidity types varied in their associations with each treatment category. For example, dementia was associated with less chemotherapy provision, however, it was not significantly related to the likelihood of surgery.

**Conclusion:**

This study indicates that the association of age with surgical treatment differed significantly by the CRC subsite. Comorbidity moderated the negative association of age with chemotherapy, and less so, with extent of surgery. Results were novel in indicating associations of multiple individual comorbidity types with CRC treatment modalities. The data suggest that different individual comorbidity types may have different effects on treatment and should be studied separately.

## Graphical abstract

Individual comorbidities associate differently with surgery and chemotherapy in colorectal cancer.

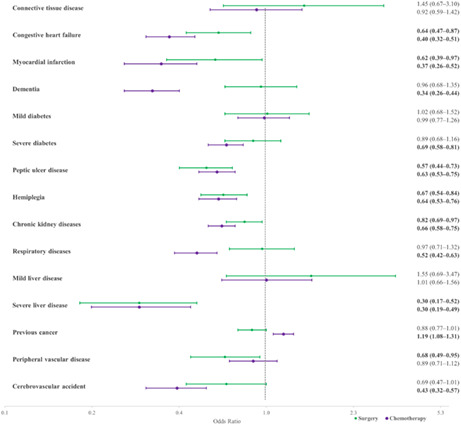

## 1 INTRODUCTION

During the last three decades, the colorectal cancer (CRC) mortality rate decreased steadily in Australia.^1^ New treatment strategies and clinical guidelines are factors that are thought to have increased the survival. However, several studies indicate that older people are less likely to get treatment for CRC.^2^, ^3^, ^4^ Such lesser treatment might partly explain why, despite older people having less advanced CRC stages, they still have poorer survival compared with younger patients.^5^, ^6^

Several factors are thought to be responsible for older people receiving less treatment, including greater frailty, difficulties with transport to treatment centers, and sometimes a preference not to be treated. It has also been suggested that comorbidities can be a reason for not offering chemotherapy, irrespective of age.^7^ Although the extent to which comorbidities impact on CRC treatment, especially among older people, has attracted limited research. Some studies found a negative association of increased comorbidity with likelihood of treatment in older people,^8^ whereas no association was found in other studies.^9^ The primary cancer site (colon or rectum) also is another determinant of treatment differences.^10^ Each site has different metastatic patterns, complications, and recurrence rates that can affect treatment selection.^11^

We used population‐based data to first investigate associations of age and other factors with pattern of care (surgery and chemotherapy) in CRC; and then, to determine how this association varied by comorbidity status and primary cancer site. Finally, we analyzed the extent to which individual comorbidities were associated with different treatments.

## 2 MATERIALS AND METHODS

### 2.1 Study population and variables

Population‐wide data on CRC diagnosed in 2004–2013 (ICD‐10: C18–C20) were extracted from the South Australian Cancer Registry (SACR). Of the 11,843 cases in the SACR dataset, 8462 (71.4%) were eligible to be included in the study, and the rest were excluded mainly due to missing data. Data items included age, diagnosis date, primary CRC site, grade, and indices of socioeconomic disadvantage and residential remoteness derived by Registry staff from residential postcodes.^12^, ^13^ Stage was provided by the South Australian Clinical Cancer Registry (SACCR) and categorized into four groups using the Australian Clinicopathological Staging System (ACPS). Charlson Comorbidity Index (CCI) was used to indicate the comorbidity burden for CRC cases, based on comorbid conditions recorded in hospital inpatient records (the Integrated South Australian Activity Collection) for the calendar year preceding cancer diagnosis.^14^ Socioeconomic status was measured using Socioeconomic Indexes for Areas (SEIFA) and categorized from most to least disadvantaged (Q1–Q5) based on Index of Relative Socio‐economic Disadvantage.^12^ Remoteness was measured based on the Accessibility/Remoteness Index of Australia, which provides a measure of the accessibility to standard services and categorized as major city, inner regional, outer regional, remote, and very remote by SACR staff using standard criteria.^13^

### 2.2 Treatment variables

Surgery and chemotherapy were recorded as received or non‐received within one year after the diagnosis of CRC. Multiple population‐based datasets were used to identify treatments for CRC patients. Colorectal surgery was identified using hospital inpatient procedure codes, Medicare Benefits Schedule (MBS) data, and whether recorded on a South Australian state‐wide metastatic CRC cancer dataset. Chemotherapy was also identified using inpatient procedure codes, MBS and Pharmaceutical Benefits Scheme (PBS) codes, and the metastatic cancer dataset. It has been demonstrated that administrative datasets have a high specificity for detecting the main treatment modalities and the sensitivity of detection increases to 97% by linking multiple datasets.^15^, ^16^, ^17^ Specific codes used for characterizing treatment modalities in different datasets are provided in the supplementary files.

### 2.3 Statistical analysis

The frequency of each treatment modality was determined for study variables. Then, the association of age and other variables with each treatment was investigated using logistic regression (adjusting for all relevant variables). For chemotherapy, we used stage B as the reference since it was rarely provided for CRC stage A.

We developed logistic regression models for treatments with age group, while restricting the model by subsite (colon or rectum respectively) and comorbidity index (CCI = 0, CCI = 1, 2, CCI >2, respectively). We examined the intensity of associations between age and treatment across primary cancer site categories and CCI levels by comparing the 95% confidence intervals.

Interaction terms were also used to indicate whether associations of age with treatments differed by subsite and comorbidity levels. Specifically, we used logistic regression models with interaction terms between age and these factors.

Logistic regression models were then developed for each chronic condition included in the CCI index to indicate associations with treatment types.

All data management and statistical analyses were performed using Stata version 16.^18^

## 3 RESULTS

A total of 8462 CRC patients in SACR dataset between 2004–2013 were included in the study. Of those, 8053 (95.2%) had evidence of recorded treatment, with surgery applying to 91.5%. The distribution of treatments by the sociodemographic and clinical features is shown in Table 1. Older age groups and those with comorbidities had smaller proportions of treated cases.

**TABLE 1:** Characteristics of CRC cases by cancer treatment received; South Australia 2004–2013.

Variable	Total (%)	Surgery (%)	Chemotherapy (%)
Age group (years)			
<50	531 (6.3)	494 (6.4)	428 (9.9)
50–59	1350 (16.0)	1282 (16.5)	930 (21.6)
60–69	2196 (25.9)	2073 (26.8)	1366 (31.7)
70–79	2643 (31.2)	2423 (31.3)	1211 (28.1)
80+	1742 (20.6)	1470 (19.9)	374 (8.7)
CCI			
0	4465 (52.8)	4189 (54.1)	2580 (59.9)
1, 2	2642 (31.2)	2396 (31.0)	1253 (29.1)
>2	1355 (16.0)	1157 (14.9)	476 (11.1)
Socioeconomic status^a^			
Q1	2260 (26.7)	2051 (26.5)	1145 (26.6)
Q2	1942 (23.0)	1776 (23.0)	982 (22.8)
Q3	1555 (18.4)	1429 (18.5)	745 (17.3)
Q4	1509 (17.8)	1377 (17.8)	785 (18.2)
Q5	1184 (14.0)	1099 (14.2)	646 (15.0)
Remoteness			
Major cities	5952 (70.3)	5438 (70.2)	3008 (69.8)
Regional areas	2170 (25.6)	1987 (25.7)	1130 (26.2)
Remote areas	340 (4.0)	317 (4.1)	171 (4.0)
Primary site			
Colon	5683 (67.2)	5222 (67.4)	2635 (61.2)
Rectum	2779 (32.8)	2520 (32.6)	1674 (38.8)
Sex			
Male	4595 (54.3)	4219 (55.5)	2427 (57.8)
Female	3705 (43.8)	3377 (44.5)	1770 (42.2)
Stage			
A	1113 (13.2)	1040 (13.4)	144 (3.3)
B	1947 (23.0)	1921 (24.8)	544 (12.6)
C	2928 (34.6)	2882 (37.2)	2093 (48.6)
D	2474 (29.2)	1899 (24.5)	1528 (35.5)
Tumor differentiation (grade)			
Well	381 (4.5)	358 (4.6)	154 (3.6)
Moderate	5480 (64.8)	5216 (67.4)	2743 (63.6)
Poor/ undifferentiated	1796 (21.2)	1695 (21.9)	1064 (24.7)
Unknown	805 (9.5)	473 (6.1)	348 (8.1)
Diagnostic period			
2004–2008	4892 (57.8)	4464 (57.7)	2308 (53.6)
2009–2013	3570 (42.2)	3278 (42.3)	2001 (46.4)

^*a*^ Sociodemographic data incomplete for 2.1% of cases.

### 3.1 Association of age with treatment

Associations of age and other variables with treatment are shown in Table S1. Ages below 80 were positively associated with receiving treatment and these associations were stronger for chemotherapy than surgery. Comorbidity was associated with not receiving these treatments. Rectal cancer patients were less likely to receive surgery (OR = 0.67, 95% CI = 0.55–0.82) and more likely to receive chemotherapy (OR = 1.68, 95% CI = 1.49–1.89) compared with patients with colon cancer. The results also showed that men are less likely than women to receive chemotherapy.

### 3.2 Associations of age with care by primary cancer site and comorbidity

Comparison of confidence intervals for associations of age with treatment by primary cancer site indicated that generally, decreasing age was associated with increasing likelihood of surgery and chemotherapy for both colon and rectal cancer. Rectal cancer patients showed a stronger association of age with surgery than colon cancer patients (Table 2).

**TABLE 2:** Association of age with care by primary site, as indicated by logistic regression; CRC cases, South Australia 2004–2013.

Reference: 80+ year	Odds ratio (95% confidence interval)	
	Colon (5572 cases)	Rectum (2717 cases)
Surgery		
<50	1.58 (0.91–2.73)	**3.55 (1.84–6.87)**
50–59	**2.66 (1.69–4.19)**	**2.83 (1.75–4.57)**
60–69	**2.14 (1.49–3.07)**	**2.89 (1.89–4.41)**
70–79	1.13 (0.85–1.52)	**2.25 (1.51–3.34)**
Chemotherapy		
<50	**20.68 (14.38–29.74)**	**17.20 (10.58–27.96)**
50–59	**14.05 (10.98–17.99)**	**14.92 (10.52–20.94)**
60–69	**11.89 (9.63–14.67)**	**12.11 (8.79–16.48)**
70–79	**5.16 (4.28–6.22)**	**5.53 (4.06–7.39)**

*Note*: Models adjusted for stage, sex, socioeconomic and remoteness status and diagnostic period. Statistically significant results shown in bold type.

Table 3 shows that associations of age with surgery is different by primary cancer site. Cases aged 70–79 years were more likely to receive surgery (relative to 80+ years group) when they had rectal cancer than colon cancer. This difference was not observed for chemotherapy.

**TABLE 3:** Association of the interaction terms between age and primary cancer site, as indicated by logistic regression; CRC cases, South Australia 2004–2013.

Reference	Interaction terms	Surgery odds ratio (95% confidence interval)	Chemotherapy odds ratio (95% confidence interval)
80+ year # colon	<50 year # Rectum	2.28 (0.98–5.30)	0.96 (0.53–1.77)
	50–59 year # Rectum	1.06 (0.55–2.03)	1.47 (0.97–2.21)
	60–69 year # Rectum	1.30 (0.75–2.25)	1.36 (0.94–1.95)
	70–79 year # Rectum	**1.85 (1.13–3.05)**	1.31 (0.92–1.86)

*Note:* Models adjusted for all study variables (see section 3.2). Statistically significant results shown in bold font.

Comparison of confidence intervals indicated that younger age was generally associated with likelihood of treatment, regardless of the comorbidity status. However, this association was weaker in the presence of elevated comorbidity (CCI >2). The extent of this difference is demonstrated in those aged 70–79 years, where the odds ratio for receiving chemotherapy for people with CCI >2 was approximately half that applying to those with no recorded comorbidity (OR = 3.43 95% CI = 2.46–4.79 vs. OR = 6.64 95% CI = 5.20–8.48) (Table 4).

**TABLE 4:** Association of age with care by comorbidity level, as indicated by logistic regression; CRC cases, South Australia 2004–2013.

Reference: (80+ year)	Odds ratio (95% confidence interval)		
	CCI = 0 (4342 cases)	CCI = 1, 2 (2600 cases)	CCI >2 (1346 cases)
Surgery			
<50	**3.20 (1.86–5.50)**	1.90 (0.86–4.16)	0.70 (0.15–3.31)
50–59	**3.61 (2.27–5.76)**	**2.16 (1.37–4.27)**	1.53 (0.89–3.42)
60–69	**3.51 (2.30–5.36)**	**1.78 (1.17–2.97)**	**1.86 (1.07–3.25)**
70–79	**2.15 (1.46–3.16)**	1.27 (0.95–2.09)	1.05 (0.67–1.64)
Chemotherapy			
<50	**20.23 (14.04–29.15)**	**22.39 (12.32–40.70)**	**24.33 (5.06–116.91)**
50–59	**17.57 (13.25–23.29)**	**18.20 (11.93–24.61)**	**6.81 (4.10–11.31)**
60–69	**13.63 (10.53–17.66)**	**14.79 (10.91–20.05)**	**6.34 (4.33–9.30)**
70–79	**6.64 (5.20–8.48)**	**5.34 (4.08–6.97)**	**3.43 (2.46–4.79)**

*Note:* Models adjusted for primary cancer site, stage, sex, socioeconomic and remoteness status, and diagnostic period. CCI, Charlson comorbidity index. Significant results shown in bold font.

Table 5 indicates that the positive association of younger age with treatments varied by comorbidity status. People aged 50–79 years were significantly less likely to receive surgery and chemotherapy (relative to those aged 80+ years) if they had comorbidities than if no comorbidity recorded (CCI = 0).

**TABLE 5:** Association of the interaction terms between age and comorbidity level with care, as indicated by logistic regression; CRC cases, South Australia 2004–2013.

Reference	Interaction terms	Surgery odds ratio (95% confidence interval)	Chemotherapy odds ratio (95% confidence interval)
80+ year # CCI = 0	<50 year # CCI = 1, 2	0.60 (0.23–1.55)	1.21 (0.60–2.43)
	<50 year # CCI >2	0.25 (0.05–1.27)	1.39 (0.27–7.14)
	50–59 year # CCI = 1, 2	0.67 (0.32–1.39)	1.15 (0.74–1.78)
	50–59 year # CCI >2	**0.38 (0.15–0.94)**	**0.47 (0.26–0.85)**
	60–69 year # CCI = 1, 2	**0.53 (0.29–0.98)**	1.29 (0.89–1.87)
	60–69 year # CCI >2	0.55 (0.28–1.07)	**0.59 (0.37–0.93)**
	70–79 year # CCI = 1, 2	0.65 (0.38–1.11)	0.88 (0.62–1.25)
	70–79 year # CCI >2	**0.47 (0.26–0.84)**	**0.59 (0.39–0.89)**

*Note:* Models adjusted for all variables (see section 3.2). Statistically significant results shown in bold font. CCI, Charlson comorbidity index (See section 3.2).

### 3.3 Individual comorbidities and pattern of care

**FIGURE 1:**
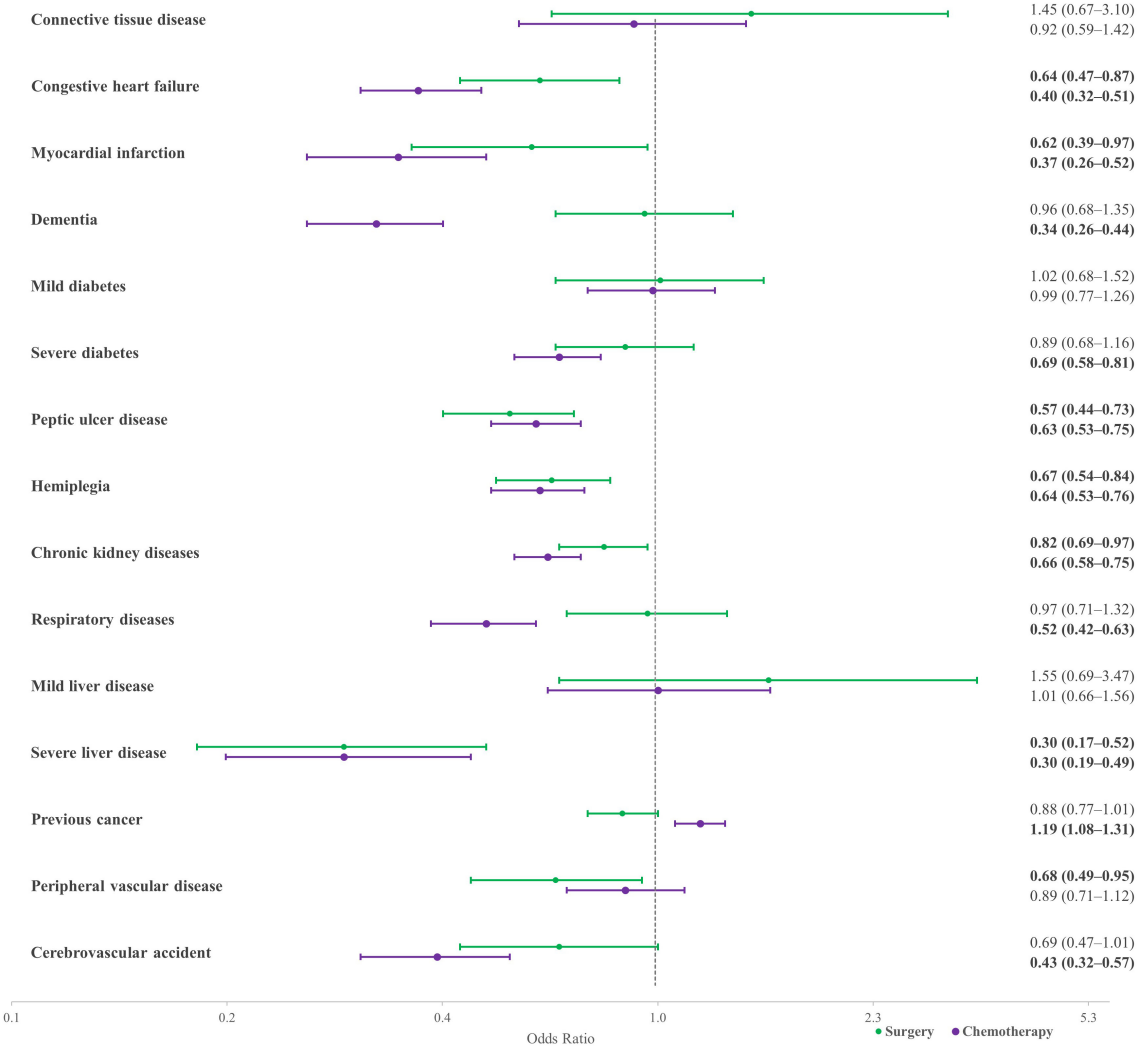
Association of comorbidities included in the CCI with surgery (green) and chemotherapy (purple), as indicated by logistic regression; CRC cases, South Australia 2004–2013. *Note:* Results adjusted for age, socioeconomic status, remoteness, primary cancer site, sex, stage, grade, and diagnostic period.

The association of individual comorbidities incorporated in CCI with treatment was explored using logistic regression models. Congestive heart failure (CHF), myocardial infarction, peptic ulcer diseases, hemiplegia, chronic kidney diseases, severe liver disease, and peripheral vascular disease were negatively associated with surgery. People who experienced cancer previously or had a history of cerebrovascular attacks were also less likely to have surgery, but the associations were not significant (OR = 0.88 95% CI = 0.77–1.01 and OR = 0.69 95% CI = 0.84–1.01 respectively). Most comorbidities, including CHF, myocardial infarction, dementia, severe diabetes, peptic ulcer disease, hemiplegia, chronic kidney disease, severe liver disease, and cerebrovascular attack were negatively associated with chemotherapy. Cases with a previous non‐CRC cancer were more likely to receive chemotherapy (OR = 1.19 95% CI = 1.08–1.31) (Figure 1).

## 4 DISCUSSION

Younger age ranges were more likely to receive treatment than people aged 80+ years. Several different reasons have been suggested in other studies, including age‐related comorbidity, and frailty.^7^, ^19^ Preference for no treatment has been raised as a main reason for less treatment of CRC, which is more common in older people.^19^, ^20^ The strength of the association between age and comorbidity with chemotherapy was stronger than surgery. Treatment guidelines in Australia do not recommend some of the chemotherapy drugs that are commonly used for CRC in people older than 65 years, such as Cetuximab or Irinotecan, either because they have not shown increased survival or are considered to be unsafe, with more adverse effects.^21^, ^22^ Also, many chemotherapy agents need to be adjusted by the glomerular filtration rate which is negatively related to age.^23^ Consequently, older people may not get the equivalent amount of effective medication compared with younger people. Increased comorbidity level was also associated with a lower likelihood of treatment in the present study. Recent studies found that patients with comorbidities were less likely to receive surgery and chemotherapy than people not so affected.^4^, ^19^, ^24^

The results showed that rectal cancer cases received less surgery than those with colon cancer, which is consistent with previous results.^25^ By comparison, rectal cancer was associated with increased likelihood of chemotherapy. This may have been influenced by Australian guidelines for CRC treatment which recommend adjuvant and neoadjuvant chemo‐radiotherapy for rectal cancer, whereas for colon cancer, only adjuvant chemotherapy is recommended.^10^

Cases with stage B and C received more surgery than stage A. We hypothesize that this may be influenced by widespread use of colonoscopic resection of stage A tumors, thereby obviating the need of further surgery. Additionally, stage D was negatively associated with receiving surgery. Many of the people with CRC stage D are not curable and will be managed by chemotherapy and palliative therapy.^26^ Cases with stages C and D were more likely in this study to receive chemotherapy in comparison to stage B. Cases with stage B CRC would only receive chemotherapy in particular circumstances (e.g., when a high risk of progression or recurrence was expected), whereas chemotherapy is a more likely to be an option for stages C and D.^10^

### 4.1 The association of age with treatment by CRC primary site and comorbidity

People aged 70–79 years were more likely to receive surgery than those aged 80+ years if the primary cancer site was rectum rather than colon. A similar picture was also observed among people younger than 50 years who had rectal cancer, although not achieving statistical significance. In other words, younger people with rectal cancer were more likely (compared to 80+ years group) to receive surgery than younger people with colon cancer. Similar analyses did not show any variations for chemotherapy provision in different age groups by the subsite of cancer.

The subgroup analysis showed that the relative odds of association between age and treatment was decreased in subgroups with higher comorbidity. For example, cases in the 70–79 age group were more likely to have surgery (compared to people 80 years and older) when they had no comorbidities. This shows that the association of age with treatment is weakened with increasing levels of comorbidity. Further analysis revealed that people in 50–79 age groups are significantly less likely to receive treatment (compared to 80+ age group) when they have comorbidities. This suggests that in people with comorbidity, age is a less decisive factor for surgery and chemotherapy provision and younger people are more likely to be treated similar to their older counterparts when they have comorbidities. Only one study has previously investigated the proportion of cases who received surgery and chemotherapy by age in different CCI status and found that in cases with CCI ≥2 the difference between age groups is no longer statistically significant.^27^ However, it should be emphasized that our results showed older people are still getting less treatment regardless of their comorbidity status.

### 4.2 Individual comorbidities have different associations with treatment

Some individual comorbidities including CHF and myocardial infarction were negatively associated with receiving cancer surgery whereas others, like diabetes or dementia, were not associated with likelihood of surgery. Severe liver disease had the strongest negative association with receiving surgery. Most chronic conditions used in the CCI were also negatively associated with receiving chemotherapy. Severe liver diseases and dementia had the strongest association with not receiving chemotherapy. A systematic review has recently revealed that dementia has a stronger association with not receiving chemotherapy in CRC compared with other comorbidities.^28^ Similar to CRC, the prevalence of dementia is expected to increase globally within the next decades.^29^ Studies have reported that people with dementia are at increased risk of lack of cancer treatment and urged the necessity to design treatment guidelines for dementia patients with cancer.^30^, ^31^ Our previous results also showed that these patients are more likely to get advanced CRC,^6^ and given the increased incidence of both disease, it is important to investigate the reason for lack of treatment in these patients and develop methods to more efficiently provide chemotherapy for these patients.

Kidney and liver diseases are expected to be negatively associated with chemotherapy provision, due to the likelihood of impaired clearance of chemotherapy agents.^32^ For example, oxaliplatin and capecitabine, which are widely used for CRC, are contraindicated for patients with renal impairment.^33^ Other chemotherapeutic agents such as bevacizumab, cetuximab, panitumumab, and regorafenib are recommended to be administered only with caution for people with comorbidities like gastrointestinal bleeding, cardiovascular disease and hepatic impairment,^22^, ^34^, ^35^, ^36^, ^37^ predisposing to reduced use. People with a previous malignancy were more likely to receive chemotherapy. This might have been due in part to continued use of a regimen that had been used for the previous cancer, which overlapped the duration of the CRC care. Other comorbidities may relate to reduced use of chemotherapy and surgery for CRC, but our study was limited to those included in the CCI. Previous studies found single comorbidities such as diabetes and cardiovascular disease to be associated with not receiving surgery and chemotherapy,^8^, ^28^ but the present study was the first to investigate associations of a range of comorbidities with CRC surgery and chemotherapy.

### 4.3 Other explanations for treatment differences

Interview surveys have indicated practitioners to be less likely to provide chemotherapy for older patients or patients with comorbidity due to wariness of complications and toxicities.^38^, ^39^ We hypothesize that treatment may not be provided because of medical concerns when weighing the value of treating the value in older people against higher risks of complications in the context of shorter remaining life expectancy. Several studies have reported adverse effects of CRC treatment among older people, including infections, toxicities, and post‐operative complications, which can reduce tolerance to treatment.^40^, ^41^, ^42^ This may have increased wariness among practitioners of treating older patients.

A number of studies have recommended particular approaches for treatment of older CRC patients in the past decade,^43^, ^44^, ^45^ and guidance has been provided for adjuvant chemotherapy of older people in Australian CRC treatment guidelines.^10^ Some international guidelines are specifically designed for the use of adjuvant therapy in older people with colon cancer, but for CRC surgery and radiotherapy of rectal cancers, little has been recommended other than geriatric assessment.^45^, ^46^, ^47^ Moreover, recommendations about the advantages of laparoscopic surgery in older people have been controversial.^47^, ^48^ This might stem from the fact that older people are often excluded from clinical trials^49^ such that limited experimental evidence is available to guide their cancer treatment. Several studies have found older patients to receive less cancer treatment, whether surgery, chemotherapy or radiotherapy.^3^, ^9^, ^50^, ^51^ It is therefore recommended to perform trials and other studies that focus on older people. This would contribute better evidence on benefits and disbenefits of CRC treatment in this population and support guideline development that is specific for this age range.^50^ In addition, facilitating transportation to hospital and improving the quality of hospital stay may encourage people to have treatment. That aside, this study indicates that socioeconomic status and remoteness were not significant factors in the treatment of CRC cases in South Australia, which we regard as positive in equity terms.

The fact that disparities in the treatment of older people varies across people with different levels of comorbidity suggests the importance of these chronic conditions in treatment decision making. This shows that a great emphasis must be placed on finding strategies and treatments that would not be limited by the presence of comorbidities. Notably, in the absence of comorbidities, a patient with advanced age may be more likely not to receive a treatment (relative to younger people) than when comorbidities exist. In addition, comorbidities are better studied individually, since they each may have different influences on likelihood of treatment. For example, while dementia is not a statistically significant correlate of surgery, it is negatively related to likelihood of chemotherapy.

### 4.4 Strengths and limitations

This study used state‐wide data from South Australia relating to CRC. The results provide evidence of variation in CRC treatment at a broad population level and reveal sociodemographic disparities.

Among limitations of this study was lack of a frailty index, which is important as this is a decisive factor in the choice of CRC treatment. Inability to identify types of chemotherapy agents was another limitation in our study. Obtaining health administrative data in Australia requires extensive scrutiny of applications through research committees, which is time‐consuming and causes a delay in reporting the results. However, the main focus of this study which is the correlation of age and comorbidity with treatment would not be significantly affected by this lack of recency.

## 5 CONCLUSION

CRC treatment guidelines are generally based on clinical trials that recruit younger patients and those with fewer comorbidities.^52^, ^53^ This can lead to inconsistency between treatment guidelines and the actual treatment of older CRC patients obtain. We demonstrated that older people with CRC were less likely to receive treatment compared with younger cases. Higher levels of comorbidity further reduce the likelihood of receiving treatment. The association of age with surgery varied significantly between colon and rectal cancer. Comorbidity moderated the association of age with chemotherapy, and to a lesser extent, surgery.

This was the first study of which we are aware of the association of multiple individual comorbidities with chemotherapy and surgery in CRC. It was apparent that different comorbidities often had different influences on treatment. Previous studies generally used only one aggregated index to measure associations with comorbidity and did not study individual comorbidities. Results also indicate the importance of using clinical trials to gain evidence of outcomes for new clinical guidelines of relevance to older patients with prevalent comorbidities.

## AUTHOR CONTRIBUTIONS

**Kazzem Gheybi:** Data curation (equal); formal analysis (equal); investigation (equal); methodology (equal); writing – original draft (equal). **Elizabeth Buckley:** Conceptualization (equal); data curation (equal); supervision (equal); writing – review and editing (equal). **Agnes Vitry:** Methodology (equal); supervision (equal); writing – review and editing (equal). **David Roder:** Data curation (equal); methodology (equal); resources (equal); supervision (lead); visualization (equal); writing – review and editing (equal).

## FUNDING INFORMATION

This study was funded by the University of South Australia.

## CONFLICT OF INTEREST STATEMENT

The authors declare that the research was conducted in the absence of any commercial or financial relationships that could be construed as a potential competing interest.

## ETHICS APPROVAL

Ethical approval for this project was granted through application to the SA Health Human Research Ethics Committees (HREC), University of South Australia's HREC and Australian Institute of Health and Welfare (AIHW) HREC. Ethics approval number: EO2016/4/317. For privacy maintenance of participants, the used data were anonymous and numbers below five where not reported. Administrative permissions were granted to the researchers (Professor Roder, Dr. Buckley, Dr. Gheybi) to access the raw data. Reference number: HREC/16/SAH/6/AM04. All methods were carried out in accordance with the guidelines and regulations in Helsinki declaration.

## Supporting information


Table S1

## Data Availability

The data used for this study are protected by the AIHW and is not publicly accessible. In order to request for access the raw data please contact Professor David Roder.
