# Supplementary material for: Variations in colorectal cancer pattern of care by age and comorbidity in South Australia
Source: Cancer Med. 2023 Apr 21;12(11):12118–27. doi: 10.1002/cam4.5901 (PMC10278522; doi:10.1002/cam4.5901)
Supplement: Supplementary file 1 — Table S1 [file CAM4-12-12118-s001.docx]

*Table 1S. Association of clinical and sociodemographic variables with treatment of CRC, as indicated by logistic regression; South Australia 2004-2013.*

| Variable (reference) | | Odds ratio for surgery (95% confidence interval) | Odds ratio for chemotherapy (95% confidence interval) |
| --- | --- | --- | --- |
| Age  (80+ year) | <50 | **2.21 (1.46-3.35)** | **18.02 (13.51-24.04)** |
|  | 50-59 | **2.56 (1.85-3.54)** | **14.06 (11.53-17.14)** |
|  | 60-69 | **2.34 (1.79-3.07)** | **11.60 (9.76-13.79)** |
|  | 70-79 | **1.47 (1.17-1.86)** | **5.22 (4.46-6.12)** |
| Charson Comorbidity index (CCI=0) | 1,2 | 0.81 (0.65-1.00) | **0.71 (0.63-0.81)** |
|  | >2 | **0.53 (0.42-0.68)** | **0.44 (0.38-0.52)** |
| Socioeconomic status  (Q=1) | Q=2 | 1.05 (0.81-1.35) | 0.97 (0.83-1.13) |
|  | Q=3 | 1.15 (0.87-1.52) | 0.94 (0.80-1.12) |
|  | Q=4 | 1.05 (0.79-1.39) | 0.99 (0.83-1.17) |
|  | Q=5 | 1.22 (0.88-1.68) | 1.01 (0.84-1.22) |
| Remoteness (major cities) | Regional areas | 0.91 (0.73-1.15) | 0.93 (0.81-1.06) |
|  | Remote areas | 1.21 (0.71-2.05) | 0.89 (0.67-1.19) |
| Primary site (Colon) | Rectum | **0.67 (0.55-0.82)** | **1.68 (1.49-1.89)** |
| Sex (male) | Female | 0.90 (0.74-1.08) | **0.83 (0.74-0.93)** |
| Stage  (stage A or B) | A | Reference | **0.29 (0.23-0.36)** |
|  | B | **5.03 (3.14-8.05)** | Reference |
|  | C | **4.10 (2.77-6.08)** | **9.26 (7.96-10.76)** |
|  | D | **0.29 (0.22-0.39)** | **7.24 (6.18-8.48)** |
| Differentiation (well) | Moderate | 1.24 (0.76-2.02) | **1.69 (1.28-2.23)** |
|  | Poor/undifferentiated | 1.34 (0.80-2.25) | **2.04 (1.52-2.73)** |
|  | Unknown | **0.17 (0.10-0.28)** | 1.02 (0.74-1.41) |
| Diagnostic period (2004-2008) | 2009-2013 | **1.58 (1.31-1.91)** | 1.05 (0.93-1.19) |

*Note: Models adjusted for all study variables. Statistically significant results shown in bold type.*

**Treatment data definition**

***Chemotherapy***

Each patient was screened in three datasets to see if they had received any chemotherapy treatment.

1. **PBS: Search for the following medications in the 12 months after diagnosis:**

- Bevacizumab: ATC code = L01XC07
- Cetuximab: ATC code = L01XC06
- Panitumumab: ATC code = L01XC08
- Fluouracil: ATC code = L01BC02
- Regorafinib: ATC code = N/A
- Oxaliplatin: ATC code = L01XA03
- Irinotecan: ATC code = L01XX19
- Capecitabine: ATC code = L01BC06
- Raltitrexed: ATC code = L01BA03

1. **MBS codes: Search for the following items in the 12 months after diagnosis:**

- 13915: CYTOTOXIC CHEMOTHERAPY, administration of, either by intravenous push technique (directly into a vein, or a butterfly needle, or the side-arm of an infusion) or by intravenous infusion of not more than 1 hours duration - payable once only on the same day, not being a service associated with photodynamic therapy with verteporfin or for the administration of drugs used immediately prior to, or with microwave (UHF radiowave) cancer therapy alone.
- 13918: CYTOTOXIC CHEMOTHERAPY, administration of, by intravenous infusion of more than 1 hour duration but not more than 6 hours duration - payable once only on the same day.
- 13921: CYTOTOXIC CHEMOTHERAPY, administration of, by intravenous infusion of more than 6 hours duration - for the first day of treatment.
- 13924: CYTOTOXIC CHEMOTHERAPY, administration of, by intravenous infusion of more than 6 hours duration - on each day subsequent to the first in the same continuous treatment episode.
- 13927: CYTOTOXIC CHEMOTHERAPY, administration of, either by intra-arterial push technique (directly into an artery, a butterfly needle or the side-arm of an infusion) or by intra-arterial infusion of not more than 1 hour duration - payable once only on the same day.
- 13930: CYTOTOXIC CHEMOTHERAPY, administration of, by intra-arterial infusion of more than 1 hour duration but not more than 6 hours duration - payable once only on the same day.
- 13933: CYTOTOXIC CHEMOTHERAPY, administration of, by intra-arterial infusion of more than 6 hours duration - for the first day of treatment.
- 13936: CYTOTOXIC CHEMOTHERAPY, administration of, by intra-arterial infusion of more than 6 hours duration - on each day subsequent to the first in the same continuous treatment episode.
- 13945: LONG-TERM IMPLANTED DRUG DELIVERY DEVICE FOR CYTOTOXIC CHEMOTHERAPY, accessing of.

1. **Hospital procedure codes for chemotherapy treatment:**

- 1393900 Loading of drug delivery device, antineoplastic agent
- 9619600 Intra-arterial administration of pharmacological agent, antineoplastic
- agent
- 9619700 Intramuscular administration of pharmacological agent, antineoplastic
- agent
- 9619800 Intrathecal administration of pharmacological agent, antineoplastic
- agent
- 9619900 Intravenous administration of pharmacological agent, antineoplastic
- agent
- 9620000 Subcutaneous administration of pharmacological agent,
- antineoplastic agent
- 9620100 Intracavitary administration of pharmacological agent, antineoplastic
- agent
- 9620200 Enteral administration of pharmacological agent, antineoplastic agent
- 9620300 Oral administration of pharmacological agent, antineoplastic agent
- 9620500 Other administration of pharmacological agent, antineoplastic agent
- 9620600 Unspecified administration of pharmacological agent, antineoplastic
- agent
- 9620700 Loading of drug delivery device, antineoplastic agent
- 9620900 Loading of drug delivery device, antineoplastic agent
- Z51.1 Pharmacotherapy session for neoplasm

**4. Surgery record in metastatic dataset**

***Surgery***

1. **Inpatient data: search for ICD-10 procedure codes:**

- 3051501 Enterocolestomy
- 3051503 Ileocolic resection with anastomosis
- 3051504 Laparoscopic ileocolic resection with anastomosis
- 3051505 Ileocolic resection with formation of stoma
- 3051506 Laparoscopic ileocolic resection with formation of stoma
- 3056500 Resection of small intestine with formation of stoma
- 3056600 Resection of small intestine with anastomosis
- 3200000 Limited excision of large intestine with formation of stoma
- 3200001 Right hemicolectomy with formation of stoma
- 3200002 Laparoscopic limited excision of large intestine with formation of stoma
- 3200003 Laparoscopic right hemicolectomy with formation of stoma
- 3200300 Limited excision of large intestine with anastomosis
- 3200301 Right hemicolectomy with anastomosis
- 3200302 Laparoscopic limited excision of large intestine with anastomosis
- 3200303 Laparoscopic right hemicolectomy with anastomosis
- 3200400 Subtotal colectomy with formation of stoma
- 3200401 Extended right hemicolectomy with formation of stoma
- 3200402 Laparoscopic subtotal colectomy with formation of Stoma
- 3200403 Laparoscopic extended right hemicolectomy with formation of stoma
- 3200500 Subtotal colectomy with anastomosis
- 3200501 Extended right hemicolectomy with anastomosis
- 3200502 Laparoscopic subtotal colectomy with anastomosis
- 3200503 Laparoscopic extended right hemicolectomy with anastomosis
- 3200600 Left hemicolectomy with anastomosis
- 3200601 Left hemicolectomy with formation of stoma
- 3200602 Laparoscopic left hemicolectomy with anastomosis
- 3200603 Laparoscopic left hemicolectomy with formation of stoma
- 3200900 Total colectomy with ileostomy
- 3200901 Laparoscopic total colectomy with ileostomy
- 3201200 Total colectomy with ileorectal anastomosis
- 3201201 Laparoscopic total colectomy with ileorectal anastomosis
- 3201500 Total proctocolectomy with ileostomy
- 3202400 High anterior resection of rectum
- 3202500 Low anterior resection of rectum
- 3202600 Ultra-low anterior resection of rectum
- 3202800 Ultra-low anterior resection of rectum with hand-sutured coloanal anastomosis
- 3203000 Rectosigmoidectomy with formation of stoma
- 3203900 Abdominoperineal proctectomy
- 3205100 Total proctocolectomy with ileoanal anastomosis
- 3205101 Total proctocolectomy with ileoanal anastomosis and formation of temporary ileostomy
- 9220800 Anterior resection of rectum, level unspecified

1. **MBS data: search for the following items in one year after diagnosis:**

- 30375: Caecostomy, Enterostomy, Colostomy, Enterotomy, Colotomy, Cholecystostomy, Gastrostomy, Gastrotomy, on a person 10 years of age or over. Reduction of intussusception, Removal of Meckel's diverticulum, Suture of perforated peptic ulcer, Simple repair of ruptured viscus, Reduction of volvulus, Pyloroplasty (adult) or Drainage of pancreas
- 30562: ENTEROSTOMY or COLOSTOMY, closure of (not involving resection of bowel), on a person 10 years of age or over
- 30563: COLOSTOMY OR ILEOSTOMY, refashioning of, on a person 10 years of age or over
- 32000: LARGE INTESTINE, resection of, without anastomosis, including right hemicolectomy (including formation of stoma)
- 32003: LARGE INTESTINE, resection of, with anastomosis, including right hemicolectomy
- 32004: LARGE INTESTINE, subtotal colectomy (resection of right colon, transverse colon and splenic flexure) without anastomosis
- 32005: LARGE INTESTINE, subtotal colectomy (resection of right colon, transverse colon and splenic flexure) with anastomosis, not being a service associated with a service to which item 32000, 32003, 32004 or 32006 applies
- 32006: LEFT HEMICOLECTOMY, including the descending and sigmoid colon (including formation of stoma)
- 32009-32057: TOTAL COLECTOMY AND ILEOSTOMY
- 32069: ILEOSTOMY RESERVOIR, continent type, creation of, including conversion of existing ileostomy where appropriate
- 32093: Endoscopic examination of the colon beyond the hepatic flexure by FIBREOPTIC COLONOSCOPY for the REMOVAL OF 1 OR MORE POLYPS, or the treatment of radiation proctitis, angiodysplasia or post-polypectomy bleeding by ARGON PLASMA COAGULATION
- 32099: RECTAL TUMOUR of 5 centimetres or less in diameter, per anal submucosal excision of
- 32102: RECTAL TUMOUR of greater than 5 centimetres in diameter, indicated by pathological examination, per anal submucosal excision of
- 32105: ANORECTAL CARCINOMA per anal full thickness excision of
- 32108: RECTAL TUMOUR, transsphincteric excision of (Kraske or similar operation)

1. **Surgery record in metastatic dataset**
